# Native valve endocarditis due to Micrococcus luteus: a case report and review of the literature

**DOI:** 10.1186/1752-1947-5-251

**Published:** 2011-06-29

**Authors:** George Miltiadous, Moses Elisaf

**Affiliations:** 1 Hippocrateon Private Hospital, Nicosia, Cyprus; 2 Department of Internal Medicine, Medical School, University of Ioannina, Ioannina, Greece

## Abstract

**Introduction:**

*Micrococcus luteus *endocarditis is a rare case of infective endocarditis. A total of 17 cases of infective endocarditis due to *M luteus *have been reported in the literature to date, all involving prosthetic valves. To the best of our knowledge, we describe the first case of native aortic valve *M luteus *endocarditis in an immunosuppressed patient in this report.

**Case report:**

A 74-year-old Greek-Cypriot woman was admitted to our Internal Medicine Clinic due to fever and malaise and the diagnosis of aortic valve *M luteus *endocarditis was made. She was immunosuppressed due to methotrexate and steroid treatment. Our patient was unsuccessfully treated with vancomycin, gentamicin and rifampicin for four weeks. The aortic valve was replaced and she was discharged in good condition.

**Conclusions:**

Prosthetic infective endocarditis due to *M luteus *is rare. To the best of our knowledge, we report the first case in the literature involving a native valve.

## Introduction

*Micrococcus *species are Gram-positive cocci that are normal inhabitants of human skin that rarely cause infectious diseases such as septic arthritis, meningitis and prosthetic valve endocarditis [1-3]. In a Medline database search of the literature the authors identified 17 previous cases of infective endocarditis due to *Micrococcus *species, all involving prosthetic valves. This particular case is of particular interest in that it is a case of infective native aortic valve endocarditis due to *Micrococcus luteus*. To the best of our knowledge, this is the first such case to be reported [4,5].

## Case presentation

A 74-year-old Greek-Cypriot woman was admitted to our Internal Medicine Clinic because of fever and malaise that had started a week previously. At three weeks prior to her admission she had undergone a total right knee replacement due to chronic osteoarthritis. Also, 10 years earlier our patient had undergone total mastectomy of the right breast and axillary lymph node dissection, due to breast cancer. Since then she had been taking tamoxifen. Additionally, seven years ago a giant cell arteritis had been diagnosed and she had been taking 15 mg of methotrexate per day and pulses of steroids. She had no recent history of dental work.

On admission, our patient was febrile (38.5°C) and tachycardic (112 beats/minute). The chest was clear to auscultation and a diastolic grade 3/6 murmur along the right sternal border was detected on cardiac examination. Clinical examination of the abdomen showed nothing remarkable. There were also no peripheral signs of infective endocarditis or neurological deficit. Finally, her recently operated right knee did not show any signs of inflammation.

Laboratory test results revealed normochromic, normocytic anemia (hematocrit 33%), leukocytosis (white blood cell count 12,000 cells/mm^3^, 80% neutrophils) and mild thrombocytosis (platelet count 415,000 cells/mm^3^). We also noted an elevated erythrocyte sedemetation rate (ESR) (110 mm/hour), and C reactive protein (CRP) (200 mg/L) and rheumatoid factor (RF) (30 IU/mL) levels. Liver function test results and serum creatine levels were within normal limits and the extracted urine sample was normal with no signs of hematuria or casts.

**Figure 1:**
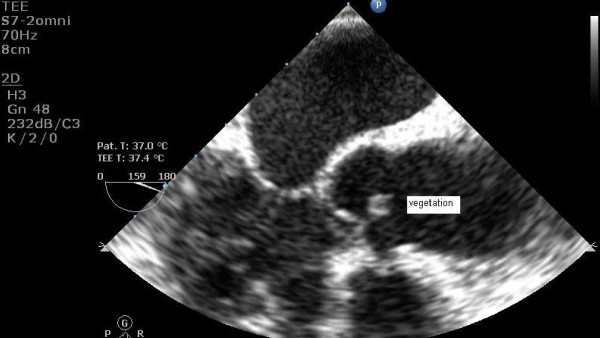
**Transthoracic echocardiography, showing a vegetation of about 1 cm on the aortic valve**.

On admission we conducted chest X-ray and upper abdomen ultasonography, the results of which were normal. An electrocardiogram (ECG) showed a right bundle branch block. Three subsequent blood cultures were drawn over a period of one hour and two of them grew *M luteus*. Transthoracic echocardiography was performed, showing a vegetation of about 1 cm on the aortic valve (Figure 1). A diagnosis of infective endocarditis was established according to the Duke criteria [6]. In fact, one major (valvular vegetation) and three minor (fever > 38°C, elevated RF and positive blood cultures of a microorganism that do not typically cause infective endocarditis) criteria were met (of note, two months earlier our patient's yearly check-up had showed normal plasma RF levels). In addition, during the course of the disease our patient had a brain embolic event. Therefore, an additional minor criterion was also met.

Our patient was treated initially with vancomycin, 2 g/daily, and intravenous gentamicin, 240 mg daily according to antimicrobial susceptibility. Due to acute renal failure, gentamicin was discontinued nine days later and replaced by 600 mg of rifampicin, After two weeks of vancomycin and rifampicin treatment our patient was still febrile up to 37.5°C. On the 30 th day of hospitalization our patient again had a high fever, up to 39°C with fever tremors, and showed dysarthria lasting about three hours. A second Transthoracic echocardiography was performed showing no differentiation. Apart from the contaminated valve no other possible sources of infection were identified through clinical examination. Our patient was then referred to the cardiac surgery department for aortic valve replacement. The biopsy of the replaced valve showed the existence of granulation tissue with signs of fibrotic repair and formation of scar tissue. Our patient was finally discharged in good health.

## Discussion

As reported by Seifert *et al*., *M luteus *is a rare cause of infective prosthetic valve endocarditis[4]. The outcome of *M luteus *endocarditis and the optimum therapeutic regimen remain to be further explored and defined. To the best of our knowledge, this is the first case ever reported concerning *M luteus *endocarditis involving a native valve. After an initial improvement, our patient experienced recurrence of septicemia (even though not documented by new blood cultures) and an embolic episode to the brain.

Our patient was immunosuppressed due to methotraxate treatment and a history of breast cancer. Furthermore, our patient had an orthopedic operation about three weeks before her admission to our clinic and, thus, a bacteremia during the procedure is a possibility. This immunosuppression and the recently performed surgery may be the causes of the infective endocarditis.

## Conclusions

To the best of our knowledge, we describe the first case of *M luteus *endocarditis involving a native valve in the present report. Therefore, clinicians should be aware of the rare possibility of *M luteus *native valve endocarditis.

## Consent

Written informed consent was obtained from the patient for publication of this case report and any accompanying images. A copy of the written consent is available for review by the Editor-in-Chief of this journal.

## Competing interests

The authors declare that they have no competing interests.

## Authors' contributions

GM was the internist in charge of our patient. ME was a major contributor in writing the manuscript. All authors have read and approved the final manuscript.
